# Disrupted Neuronal Dynamics of Reward Encoding in the Medial Prefrontal Cortex and the Ventral Tegmental Area after Episodic Social Stress

**DOI:** 10.1523/ENEURO.0229-25.2025

**Published:** 2025-07-24

**Authors:** Hannah Harris, Avery Woods, Yixin Chen, Alberto Del Arco

**Affiliations:** ^1^Department of Biomolecular Sciences, School of Pharmacy, University of Mississippi, Oxford, Mississippi 38677; ^2^Computer and Information Science, University of Mississippi, Oxford, Mississippi 38677; ^3^HESRM, School of Applied Sciences, University of Mississippi, Oxford, Mississippi 38677; ^4^Department of Psychiatry and Human Behavior, School of Medicine, University of Mississippi Medical Center, Jackson, Mississippi 39216

**Keywords:** neuronal population, rat, reward-seeking behavior, single neurons, sustained activity, vulnerability

## Abstract

Previous research suggests that stress predisposes individuals to develop substance use disorders by disrupting the brain processing of rewards. Yet, how stressful experiences disrupt the brain processing of reward-related cues at the neuronal level is poorly understood. Intermittent social defeat (ISD) is a stress animal model that increases reward-seeking behavior, drug self-administration, and choice impulsivity up to several weeks after stress. We tested the hypothesis that ISD disrupts the neuronal encoding of reward cues in key areas of the brain that regulate reward-seeking. We examined in vivo neuronal dynamics in response to reward cues in the dorsal medial prefrontal cortex (dmPFC) and the ventral tegmental area (VTA) simultaneously, and longitudinally, in control and stressed Long–Evans male rats during a discriminative stimulus reward-seeking task. In the dmPFC, ISD decreased cue-evoked neuronal activity 1 and 15 d after stress, which indicates a long-term degradation of outcome anticipation-related processing. In the VTA, ISD increased cue-evoked neuronal activity 1 d after stress but decreased cue-evoked activity 15 d after stress. Moreover, decoding analysis in single neurons and populations showed parallel increases and decreases in reward discrimination accuracy in the VTA which points to time-dependent changes in incentive salience after stress. These results demonstrate that ISD differently disrupts the neuronal encoding of reward cues in the dmPFC and the VTA and identify novel neurofunctional signatures that underlie a higher predisposition to seek out rewards after stress.

## Significance Statement

Despite the well-established relationship between stress and higher predisposition to develop substance use disorders, we still do not understand how stressful experiences disrupt the brain processing of reward cues at the neuronal level. Unlike previous studies, here we examine cue-evoked neuronal activity in the dmPFC and the VTA simultaneously and longitudinally in a well-established animal model of stress-induced vulnerability to substance abuse. We find a disrupted neuronal encoding of reward cues that span single neurons and neuronal populations and depends on brain area and time passed after stress. These results are consistent with dynamic alterations in outcome anticipation-related processing and salience attribution and provide novel insights into the neurofunctional mechanisms that drive the transition from stress to substance abuse.

## Introduction

Stressful life experiences predispose individuals to developing substance use disorders (Piazza and Le Moal, 1996; Sinha, 2001; Miczek et al., 2008; Mantsch et al., 2016; Koob and Schulkin, 2019). Clinical and preclinical studies suggest that stress-induced predisposition is driven by alterations in the brain processing of reward-related cues (Beckmann et al., 2011; Courtney et al., 2015; Volkow and Morales, 2015; Guillem and Ahmed, 2017; Novick et al., 2018; Zilverstand et al., 2018; Koban et al., 2023). Yet, how stressful experiences disrupt the processing of reward-related cues at the neuronal level is poorly understood. Cue-evoked neuronal activity in both the dorsal medial prefrontal cortex (dmPFC) and the ventral tegmental area (VTA) encodes reward-related features (i.e., outcome anticipation, salience attribution) that are critical for value-based decision-making and context-dependent reward-seeking behavior (Robinson et al., 2013; Moorman and Aston-Jones, 2015; Simon et al., 2015; Kim et al., 2016; Del Arco et al., 2017; Otis et al., 2017; Park and Moghaddam, 2017; Halbout et al., 2019; Riaz et al., 2019; Van Zessen et al., 2021), which suggests that stressful experiences disrupt the neuronal encoding of reward cues in the dmPFC, the VTA, or both. Our study tests this hypothesis by recording and comparing cue-evoked neuronal activity in the dmPFC and the VTA during a reward-seeking task in a well-established animal model of stress-induced vulnerability to substance abuse (Miczek et al., 2008; Montagud-Romero et al., 2018; Vannan et al., 2018).

Previous research investigates the effects of chronic stress on neuronal activity in the dmPFC (Kumar et al., 2014; McKlveen et al., 2015; Moda-Sava et al., 2019; Barthas et al., 2020; Spring et al., 2021a) and the VTA (Krishnan et al., 2007; Cao et al., 2010; Tye et al., 2012; Chaudhury et al., 2013; Hollon et al., 2015; Douma and de Kloet, 2020) and associate the effects of stress with changes in reward-related behavior. However, these studies are limited in two ways; first, they assess basal, but not cue-evoked, neuronal activity changes, and second, they focus on deficits in reward motivation (i.e., anhedonia) and depression-like behaviors, which are behavioral impairments typically observed after chronic stress protocols. Unlike previous studies, our study utilizes intermittent (episodic) social defeat (ISD) stress. ISD does not lead to depression-like behaviors and produces opposite effects on motivation compared with chronic (continuous) social defeat stress (Miczek et al., 2011) and increases reward-seeking behavior and drug self-administration up to several weeks after the last stress episode (Miczek et al., 2011; Boyson et al., 2014; Holly et al., 2015; Yap et al., 2015; Lemon and Del Arco, 2022). Moreover, our recent research shows that ISD increases choice impulsivity (Martinez et al., 2024), which is a value-based decision-making deficit that anticipates the escalation to substance abuse in both humans and experimental animals (Amlung et al., 2019). Therefore, we argue that ISD represents an optimal model to identify reliable neuronal signatures of reward processing that predict the transition from stress to addictive behaviors (Tunstall and Carmack, 2016).

We hypothesized that ISD disrupts neuronal dynamics in response to reward cues in both the dmPFC and the VTA during a discriminative stimulus-driven reward-seeking task (DS task). By using the DS task, previous studies have shown that contextual cues guide reward-seeking behavior and that dmPFC and VTA encode both reward and nonreward cues (Yun et al., 2004; Ishikawa et al., 2008; Moorman and Aston-Jones, 2015; Spring et al., 2021b). Since both areas of the brain play complementary roles in encoding reward cues (Volkow et al., 2017; Laubach et al., 2021), we recorded simultaneously and compared neuronal activity in the dmPFC and the VTA 1 and 15 d after stress. We have previously shown that ISD can produce different short- and long-term effects on reward-related behaviors (Sullivan et al., 2019; Lemon and Del Arco, 2022). In addition, we utilized decoding analysis to assess reward value discrimination accuracy in single neurons and populations from control and stressed animals. We find that ISD differently alters both cue-evoked neuronal activity and discrimination accuracy in the dmPFC and the VTA suggesting a disrupted neuronal encoding related to outcome anticipation and salience attribution. These effects span single neurons and neuronal populations and were dependent on time passed after stress.

## Materials and Methods

### Animals

Sixteen adult male Long–Evans rats (Inotiv; 325–375 g) were pair-housed in standard polycarbonate cages (45 × 24 × 20 cm) on a 12 h light/dark cycle (lights on at 9:00 P.M.); thus, our findings are limited to males. The sample size was based on our previous work (Del Arco et al., 2020; Lemon and Del Arco, 2022). In addition, four males (retired breeders; 500–600 g) and four females (300–325 g; Long–Evans) were utilized as residents during the stress protocol (see below). All experiments were performed during the dark phase when the animals are most active. The rats (except the residents) were placed on a mild food-restricted diet (15 g of chow per rat and day) 2 d before starting behavioral experiments (Del Arco et al., 2017; Lemon and Del Arco, 2022). All procedures were approved by the University of Mississippi Institutional Animal Review Board and were conducted in accordance with the National Institute of Health Guide for the Care and Use of Laboratory Animals.

### Experimental design

**Figure 1.**
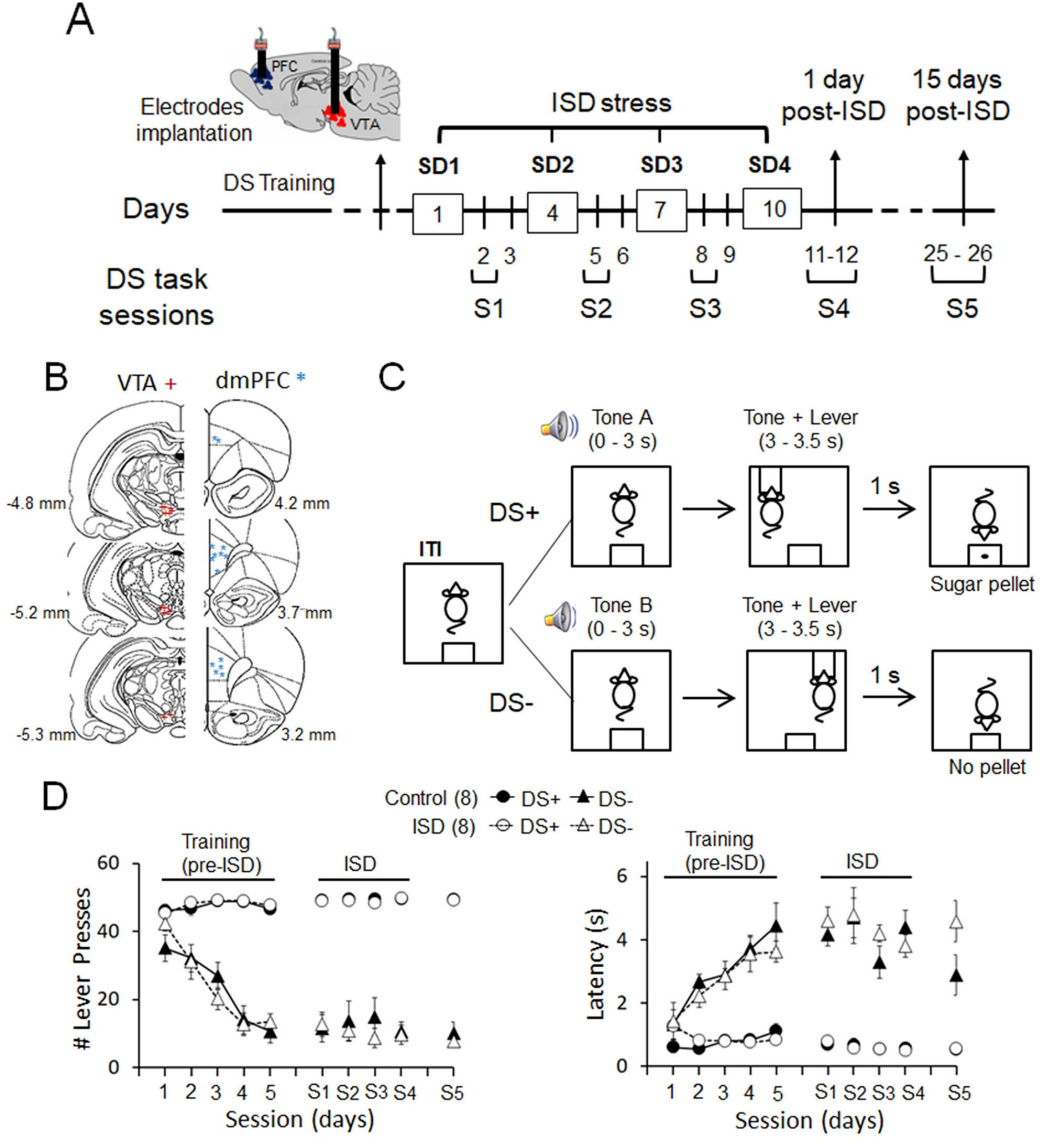
Experimental protocol, electrode placement, and DS task performance. ***A***, After training in the DS task, animals were implanted with electrode arrays in the dmPFC and the VTA and then exposed to ISD (*n* = 8 rats; or handling, control animals, *n* = 8 rats). Behavior (S1–S5) and recording sessions (S4 and S5) were carried out the days in between social defeat episodes and after ISD. ***B***, Representation of the histological placement of electrodes in the dmPFC and the VTA. ***C***, Diagram illustrating the DS task protocol used in this study. ***D***, Graphs represent the number of lever presses (mean ± SEM) and latency (mean ± SEM) during the training sessions (1–5, before ISD) and after starting ISD stress (and handling; S1–S5). Neuronal recordings were carried out during sessions S4 (1 d post-ISD) and S5 (15 d post-ISD). During the training sessions, animals learned to discriminate DS+ against DS− reflected by a decrease in the number of lever presses (trial type × session: *F*_(4,40)_ = 31.25, *p* < 0.001, three-way ANOVA) and an increase in latency (trial type × session: *F*_(4,24)_ = 8.78, *p* < 0.001) in DS− trials across sessions. ISD did not change DS task performance during or after ISD.

Two weeks after their arrival at the animal facility, all rats were handled, for at least 3 d, and then habituated to the operant chambers and trained in the discriminative stimulus reward-seeking task (DS task). Once animals achieved stable performance, electrodes arrays were implanted in the dmPFC and the VTA trough stereotaxic surgery. Immediately after surgery, animals were single-housed and remained single-housed for the duration of the study. One week after surgery, animals were retrained in the DS task to criterion performance and then divided into two groups: Control (*n* = 8) and Stress (*n* = 8). The Stress group was exposed to intermittent social defeat (ISD) following the protocol used in our previous work (Sullivan et al., 2019; Lemon and Del Arco, 2022; Martinez et al., 2024) and depicted in Figure 1*A*. The Control group was taken to a different room for the same amount of time and handled for 5 min. Rats were tested in the DS task 24 h after each stress episode, and electrophysiological recording sessions were performed 1 and 15 d after the end of the stress protocol. After completion of experiments, animals were anesthetized and their brains removed to confirm electrode's placement in the dmPFC and the VTA (Fig. 1*B*).

### Behavioral task

Figure 1*C* depicts the DS task. We modified this task from Moorman and Aston-Jones (2015). The apparatus consisted of a sound-attenuated operant chamber with two retractable levers (right and left) and a food trough in between them on the same wall (Coulbourn Instruments). The chamber contained a house light that was on during the entire session. First, animals were trained to establish the lever–response contingency through a reward-shaping procedure using both levers (3–4 d, 30 min sessions; dustless sugar pellets, 45 mg; Bio-Serv). Then, animals were trained in the DS task. In this task, an active or an inactive lever was associated with a unique tone (active, continuous 2 kHz tone; inactive, intermittent 2 kHz tone; 60–65 dB) forming a reward (DS+) and a nonreward (DS−) cue (speaker box, Coulbourn Instruments). Active and inactive levers were presented individually in every trial in a pseudorandom fashion and remained the same for an individual animal but were different for each animal. Tones were played for 3 s before the lever presentation and a maximum of 3.5 s. The levers were extended 3 s after the tone and remained extended for a maximum of 10 s. Reward cue lever presses resulted in tone offset, lever retraction, and the illumination of the food trough indicating that a food pellet dropped. Nonreward lever presses did not have any consequence (no pellet) other than tone offset and lever retraction. If the rat did not respond to the lever after 10 s, the lever retracted, and the trial was counted as an omission. The intertrial interval was set at 30 s. Animals performed until completing 100 trials, 50 DS+ and 50 DS− trials, distributed pseudorandomly through the task. DS training lasted until animals pressed the nonreward lever <20% of trials for at least two consecutive sessions.

### Surgery and electrophysiology procedure

Chronic microelectrode arrays were implanted under isoflurane anesthesia in the dmPFC (prelimbic; AP = +3.0, *L* = 0.7, *V* = −4, from bregma) and the VTA (AP = −5.3, *L* = 0.5–1.1, *V* = −8.3, from bregma) of rats (Del Arco et al., 2017, 2020). Microelectrode arrays consisting of eight polyimide-insulated Tungsten wires (50 mm) made in-house were implanted in the VTA. Microelectrode arrays consisting of 16 Teflon-insulated stainless-steel wires (50 mm; NB Labs) were implanted in the dmPFC. Electrode arrays were secured onto the skull with dental cement using six screws as anchors. A silver wire was connected to one of the screws used as a ground. Single units were recorded by a unity-gain field-effect transistor head stage and lightweight cabling, which passed through a commutator (Plexon) to allow freedom of movement in the test chamber. Recorded neuronal activity was amplified at 1,000× gain and digitized at 40 kHz by the recorder software (Plexon). Single-unit activity was digitally high-pass filtered at 300 Hz, and local field potentials were low-pass filtered at 125 Hz. Event markers from the operant box were sent to the recorder to synchronize behavior events of interest (cue onset, lever presentation) with neuronal activity. Single units were isolated in Offline Sorter (Plexon) using a combination of manual and semiautomatic sorting techniques (Del Arco et al., 2017, 2020).

### Intermittent social defeat stress

After electrodes implantation and stable performance in the DS task, rats were exposed to ISD as in our previous work (Resident–Intruder paradigm; Lemon and Del Arco, 2022; Martinez et al., 2024). Resident males (Long–Evans retired breeders; 500–600 g) were housed in transparent PVC cages (*H* × *L* × *W*: 45 × 61 × 61 cm) with females for at least 10 d before starting the procedure. Female rats were previously sterilized by ligation of the oviducts (Inotiv). Animals of the Stress group were exposed to social defeat once every 3 d for 10 d (Fig. 1*A*). Control animals were moved to a new room the same amount of time and handled for 5 min. Every social stress session started by removing the female rat from the resident cage at least 30 min before. Then, first, the intruder rat was placed in the cage with the resident male separated by a divider wall that contained wire mesh (allowing sensory exposure)—both rats could see and smell each other, for 10 min. Second, the divider wall was removed, allowing the rats to physically interact. The interaction was stopped when either six attacks were witnessed, the intruder was in supine position for 5 s, or 5 min had elapsed. To avoid injuries, the interaction was also stopped if an aggressive bite occurred. The latency to the first attack and the time in supine position were recorded. Third, the divider wall was reinserted, and the intruder remained in the cage for 10 more minutes. After this time, intruders returned to their home cages. Intruders were not exposed to the same resident more than twice.

### Histology

After completion of the experiment, rats were deeply anesthetized [ketamine (100 mg/kg)/xylazine (15 mg/kg) mixture, i.p.] and then perfused with saline and 10% formalin. The brains were then removed and kept refrigerated. Brains were sectioned in coronal slices, stained with cresyl violet, and mounted to microscope slides. Electrode-tip placements were examined under a light microscope. Only rats with correct placements within the dmPFC and the VTA were included in electrophysiological analyses (Fig. 1*B*).

### Analysis of electrophysiological data

Electrophysiological data were analyzed with custom-written scripts, executed in MATLAB (MathWorks), along with the Chronux toolbox (http://chronux.org/). The activity of each unit (spikes/s) during DS+ and DS− trials was averaged in 50 ms bins and normalized (*z*-score) across time from −1 s to +4 s around the cue onset (100 bins of 50 ms). Then, we computed 200 ms windows advancing in 50 ms to identify units that were responsive to cue and lever presentation events. First, units were classified as activated or inhibited by these events for each 200 ms window if their average absolute activity was *Z* > 2 or *Z* < −2, respectively. Then, based on previous studies (Tindell et al., 2005; Ferguson et al., 2020; Munkhzaya et al., 2020; Goedhoop and Arbab, 2023), we split the tone period (0–3 s) into tone rapid (Tone^R^, 0–0.4 s) and tone sustained (Tone^S^, 0.5–3 s), to evaluate both immediate and slow/sustained neuronal activity changes, respectively, in response to the cue. For the analysis of the lever presentation (3 s), we focused on the first 0.4 s counting from the presentation of the lever (Lever, 3–3.4 s). To be considered responsive, a unit had to display significant firing rate changes (compared with baseline) in at least 1 bin (200 ms bins) from 0 to 0.4 s for Tone^R^, 6 bins from 0.5 to 3 s for Tone^S^, and 1 bin from 3 to 3.4 s for Lever. The baseline period for Tone^R^ and Tone^S^ was from −1 to 0 s, and for Lever was from 2 to 3 s, with respect to the cue onset. The basal firing rate of each unit was calculated as the average activity from −1 to 0 s using all trials. Putative dopamine (DA) and nondopamine (non-DA) units were identified using the firing rate (<12 Hz for DA) and waveform duration (<1.2 ms for DA) as criteria (Kim et al., 2010; Park and Moghaddam, 2017; Del Arco et al., 2020). The DA waveforms patterns were consistent with optogenetically identified DA neurons in the VTA of the same strain of rats (Lohani et al., 2019).

The area under the receiver operating characteristic (auROC) curve was computed in 50 ms bins to identify the units that were selective discriminating between reward and nonreward cues. auROC was bounded by 0 and 1, with more extreme ROC values indicative of greater discrimination, and 0.5 indicative of no discrimination, between conditions (Wallis and Miller, 2003; Del Arco et al., 2017). A unit was considered selective during Tone^R^, Tone^S^, and Lever, if the average of 50 ms auROC values across the corresponding time periods was significantly different from the baseline (*Z* > 2). For each unit, we shuffled DS+ and DS− trials and recalculated auROC 10 times. The proportion of selective units was compared with averaged shuffle values to determine statistical significance. auROC increases (Δ auROC) during Tone^R^, Tone^S^, and Lever were calculated as the average of 50 ms auROC values across the corresponding time periods after subtracting baseline auROC values.

For decoding analysis, we utilized machine learning algorithms previously applied to neuronal data (Siciliano et al., 2019; Glaser et al., 2020) and specifically the regression logistic model LASSO (Least Absolute Shrinkage and Selection Operator) due to its ability to select the features that were most useful to the model's prediction (Akalin, 2020; Koban et al., 2023). Decoding accuracies were computed considering the 10 most frequent features selected by the model. First, we prepared the neuronal activity dataset. For each cell at a given trial, the population activity was aligned around the cue onset time and extended to 1 s after the lever presentation (from −1 to 4 s). Second, we used time stamps of average population activity for each trial as features to discern when neuronal populations better predicted the value of the cue. Then, each trial was given a label based on the value of the cue (DS+ or DS−). We used a binary labeling system, 1 for DS+ and 0 for DS−. We then combined data across all animals and trials to construct a full dataset with *n* features (100 features = 100 of 50 ms time bins) and *n* labels, where *n* was the number of all the trials across animals in each session (50 DS+ and 50 DS− trials per animal). Third, we split our full dataset into a training set and testing sets. We then optimized the hyperparameters of the decoder using a fivefold cross-validation within the training set. After optimization, we retrained the decoder based on the full training set. Finally, we applied the trained model on testing sets to obtain decoding accuracy. We repeated this process, using different training sets, 100 times and then selected the 10 most frequent features to calculate mean accuracy during cue and lever presentation events in the DS task and statistically compare Control and Stress groups.

### Statistical analysis

Behavioral performance was analyzed with a three-way ANOVA considering trial type (DS+ vs DS−) and session (1 vs 15 d) as within subjects and group (Control vs ISD) as between subject's variables. Single units were analyzed with chi-squared and paired Student’s *t* tests to identify differences in the proportion of units and auROC values, respectively. The Fisher exact test was used to compare the proportion of units with small sample sizes (*n* < 5). Independent Student’s *t* tests were used to compare basal firing rates, average population activity of units and decoding accuracy between groups. Welch's *t* test was utilized when sample sizes were different and/or the equality of variances was not presumed. Effect sizes were estimated through the phi coefficient after chi-squared and the Cohen's *d* after *t* tests. *p* < 0.05 was considered the cutoff for statistical significance.

## Results

### Discriminative stimulus-driven reward-seeking behavior

We utilized a discriminative stimulus reward-seeking task (DS task; modified from Moorman and Aston-Jones, 2015; Fig. 1*C*) to investigate whether ISD disrupts neuronal encoding and discrimination of reward cues. Figure 1*D* shows that, before ISD, all rats (*n* = 16) learned to discriminate reward (DS+) from nonreward (DS−) cues in five training sessions in which they performed 100 trials (50 DS+ and 50 DS−, pseudorandom) per session (day). Across sessions, the rats pressed the lever associated with DS+ more times (48.25 ± 0.48) compared with DS− (13.72 ± 2.24, average of the last two training sessions; trial type × session: *F*_(4,40)_ = 31.25, *p* < 0.001, three-way ANOVA). Stimulus discrimination learning was also reflected in the time that the rats took to press both DS+ and DS− levers (i.e., Latency). The latency (seconds) to press the DS+ lever was lower (0.88 ± 0.08) compared with the latency to press the DS− lever (3.76 ± 0.28, average of the last two training sessions; trial type × session: *F*_(4,24)_ = 8.78, *p* < 0.001; Fig. 1*D*). There were no differences in the number of lever presses (trial type × session × group: *F*_(4,40)_ = 1.75, *p* = 0.158) or latencies (trial type × session × group: *F*_(4,24)_ = 0.26, *p* = 0.897) between the Control and the Stress group during training and before starting the ISD protocol.

After training, the rats in the Stress group (*n* = 8) were exposed to ISD and the rats in the Control group (*n* = 8) were exposed to handling (see Materials and Methods). Then, both Control and Stress groups were tested in the DS task in the days between stress episodes (S1–S3) and 1 d (S4) and 15 d (S5) after the last stress episode (Fig. 1*A*) to assess whether there were time-dependent differences in the effects of ISD on DS task performance. As shown in Figure 1*D*, ISD did not significantly change the number of lever presses (trial type × session × group: *F*_(4,52)_ = 0.47, *p* = 0.758) or latency (trial type × session × group: *F*_(4,40)_ = 0.17, *p* = 0.954) during DS+ or DS− trials across sessions (i.e., S1–S5). These results indicate that ISD does not alter the ability of animals to discriminate reward cues and earn sugar pellets during the DS task.

### dmPFC and VTA neurons differently respond to reward and nonreward cues

**Figure 2.**
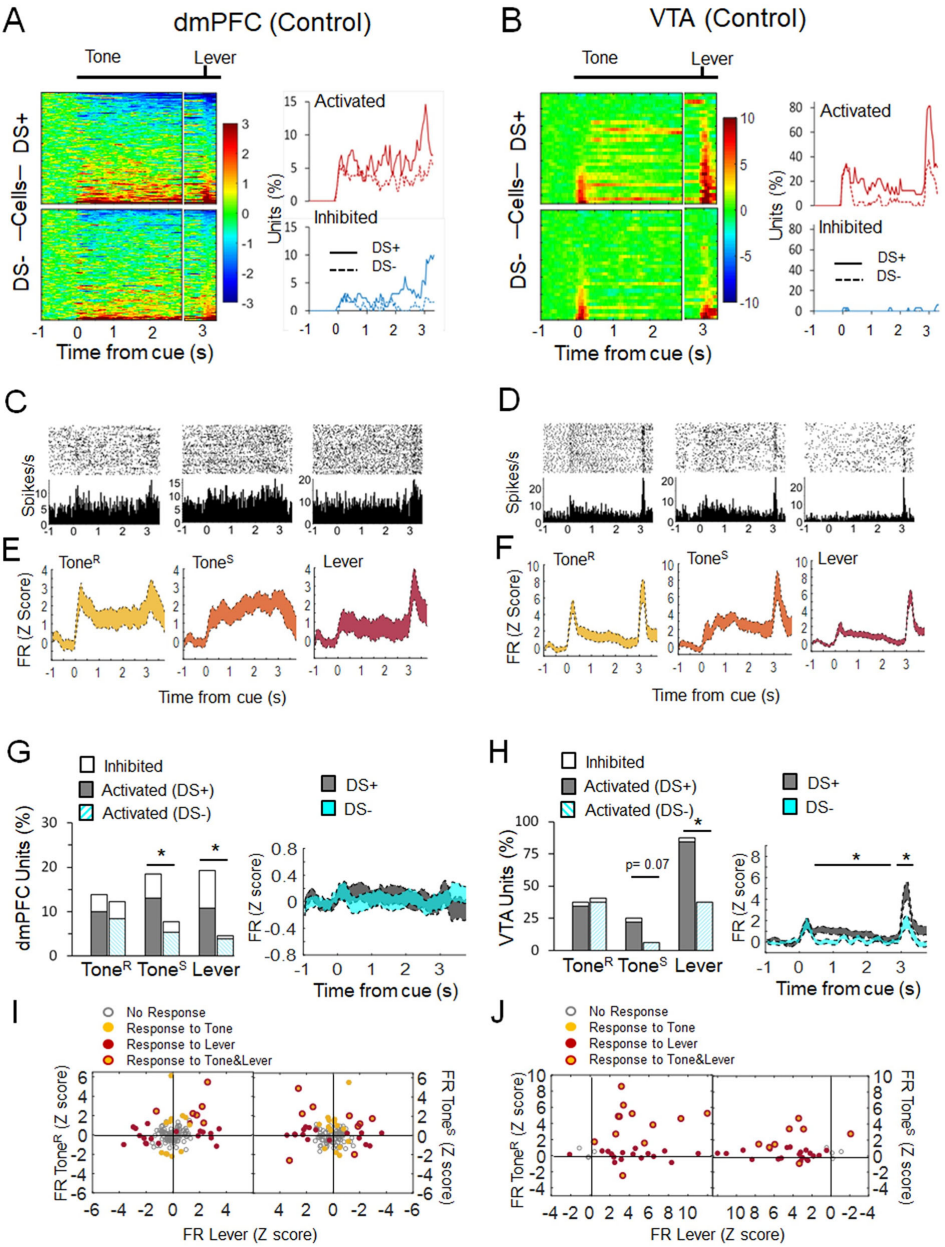
dmPFC and VTA neurons differently respond to reward and nonreward cues during the DS task. ***A***, ***B***, Heat plots represent changes in the baseline-normalized firing rate (*z* scores) for each unit from dmPFC (***A***, *n* = 130 units) and VTA (***B***, *n* = 32 units) in response to the cue (Tone) and lever presentation events (*n* = 6–7 rats). Each row is the average activity of a single unit in 200 ms windows that advanced in 50 ms steps from −1 to 3.5 s around the cue onset. Rows are aligned to corresponding task events and sorted from lowest to highest average firing rate. The line graphs are the percentage of neurons activated and inhibited by these events during DS+ and DS− trials. Units were categorized as responsive, activated, or inhibited, over time based on whether their averaged activity by 200 ms time windows was significantly different from baseline activity. ***C***, ***D***, Peristimulus histogram and spike raster plot examples of representative units responding during Tone^R^ (0–0.4 s), Tone^S^ (0.5–3 s), and Lever (3–3.4 s) in the dmPFC (***C***) and the VTA (***D***). ***E***, ***F***, Average of the population of responsive units activated during Tone^R^, Tone^S^, and Lever in the dmPFC (***E***) and the VTA (***F***). ***G***, ***H***, Bar graphs represent the percentage of units significantly activated and inhibited during Tone^R^, Tone^S^ and, Lever in DS+ and DS− trials. In the dmPFC (***G***), there were more units responding to Tone^S^ (*χ*^2^_(2)_ = 6.63, *p* = 0.036) and Lever (*χ*^2^_(2)_ = 14.17, *p* < 0.001), but not Tone^R^ (*χ*^2^_(2)_ = 0.18, *p* = 0.912), in DS+ compared with DS− trials. In the VTA (***H***), there were more units responding to Lever (*χ*^2^_(1)_ = 14.76, *p* < 0.001), but not Tone^R^ (*χ*^2^_(1)_ = 0.06, *p* = 0.794) or Tone^S^ (*χ*^2^_(1)_ = 3.23, *p* = 0.072), in DS+ compared with DS− trials. Line graphs represent the average population activity (mean ± SEM). In the dmPFC, there were no differences between DS+ and DS− trials. In the VTA, the population activity was higher during DS+ compared with DS− trials in response to Tone^S^ (*t*_(31)_ = 3.56, *p* = 0.001) and Lever (*t*_(31)_ = 4.62, *p* < 0.001, Welch's *t* test). ***I***, ***J***, Representation of single units according to their response to Tone^R^, Tone^S^, and/or Lever in the dmPFC (***I***) and the VTA (***J***). The response is the average normalized (*z*-score) firing rate (FR) during these task events. The graph highlights the units that respond to more than one task event.

We first analyzed how neurons encoded reward and nonreward cues in the dmPFC and the VTA during the DS task in control conditions (Table 1, Fig. 2). In both DS+ and DS− trials, we examined neuronal responses during the tone (0–3 s) and lever presentation (3 s) events, since both events anticipated the possibility of earning rewards with different proximity. Based on previous studies (Tindell et al., 2005; Ferguson et al., 2020; Munkhzaya et al., 2020; Goedhoop and Arbab, 2023) and our own observations, we split the tone period into tone rapid (Tone^R^, 0–0.4 s) and tone sustained (Tone^S^, 0.5–3 s), to evaluate both immediate and slow/sustained neuronal activity changes, respectively, in response to the tone. For the analysis of the lever event, we evaluated the first 0.4 s counting from the presentation of the lever (Lever, 3–3.4 s) and therefore focused on neuronal responses before animals press the lever (Fig. 1*D*, Latency).

**Table 1.** Proportion of responsive units activated, and inhibited (in parenthesis), during the tone and lever events in both DS+ and DS− trials in the dmPFC and the VTA [putative DA/non-DA cells] of control and stressed animals 1 d post-ISD

Control	dmPFC		VTA	
	DS+	DS−	DS+	DS−
Tone^R^	10% (4%)	8% (4%)	34% (3%) [34%/3%]	38% (3%) [38%/3%]
Tone^S^	13% (5%)*	5% (2%)	22% (3%) [22%/3%]	6% (0%) [6%/0%]
Lever	11% (8%)*	4% (1%)	84% (3%)* [75%/13%]	38% (0%) [34%/3%]
ISD				
Tone^R^	1% (1%)**	4% (3%)	60% (4%)** [49%/14%]	70% (4%) [53%/19%]
Tone^S^	3% (5%)**	3% (1%)	33% (0%)* [23%/11%]	5% (0%) [5%/0%]
Lever	11% (7%)*	4% (0%)	74% (9%)* [60%/23%]	37% (14%) [33%/18%]

* *p* < 0.05 compared with DS−.

** *p* < 0.05 compared with Control.

We recorded 495 neurons in the dmPFC (248 units, Control, *n* = 7; 217 units, ISD, *n* = 7) and 152 neurons in the VTA (56 units, Control, *n* = 6; 96 units, ISD, *n* = 6), in two DS task sessions, session 4 (1 d post-ISD) and session 5 (15 d post-ISD; units per rat dmPFC: Control: 39, 45, 47, 35, 11, 42, 29; ISD: 10, 33, 47, 14, 33, 22, 58. units per rat VTA: Control: 5, 2, 2, 13, 32, 2; ISD: 17, 3, 24, 17, 3, 32). The electrode arrays from two animals in the dmPFC (1 control and 1 ISD) and four animals in the VTA (2 controls and 2 ISD) did not work and therefore the data from these animals could not be used for the analysis. Figure 2 shows neuronal activity changes during session 4 in control animals (130 units, dmPFC and 32 units, VTA) locked to tone and lever presentation events.

In the dmPFC, neurons were responsive, activated, and inhibited, during Tone^R^, Tone^S^, and Lever in both DS+ and DS− trials (Fig. 2*A*,*C*,*E*,*G*,*I*; see Materials and Methods). Figure 2*G* shows that a different proportion of units were responsive in DS+ compared with DS− trials during Tone^S^ (*χ*^2^_(2)_ = 6.63, *p* = 0.036) and Lever (*χ*^2^_(2)_ = 14.17, *p* < 0.001), but not Tone^R^ (*χ*^2^_(2)_ = 0.18, *p* = 0.912). It also shows that the average population activity was not different in DS+ compared with DS− trials. Figure 2*I* represents the firing rate changes of responsive single units during DS+ trials and shows that only some units were responsive to more than one task event. Specifically, one third of units responding during Tone^R^ (6 out of 18; 33%) and Tone^S^ (9 out of 24; 37%) also responded to Lever. In the VTA, most neurons were activated during Tone^R^, Tone^S^, and/or Lever in DS+ and DS− trials (Fig. 2*B*,*D*,*F*,*H*,*J*). Figure 2*H* shows that more VTA neurons were responsive in DS+ compared with DS− trials during Lever (*χ*^2^_(1)_ = 14.77, *p* < 0.001), but not Tone^R^ (*χ*^2^_(1)_ = 0.06, *p* = 0.794) or Tone^S^ (*χ*^2^_(1)_ = 3.23, *p* = 0.072). The average population activity was also higher in DS+ compared with DS− trials during both Tone^S^ (*t*_(31)_ = 3.56, *p* = 0.001, Welch's *t* test) and Lever (*t*_(31)_ = 4.62, *p* < 0.001, Welch's *t* test). Unlike dmPFC, VTA units responding to Tone^R^, Tone^S^, and Lever were highly overlapped (Fig. 2*J*). Specifically, 100% of units responding during both Tone^R^ and Tone^S^ also responded to Lever. Overall, these data indicate that the same population of neurons in the VTA, but not in the dmPFC, encode cue and lever presentation events and that both dmPFC and VTA neurons are more responsive to reward (DS+) compared with nonreward (DS−) cues, during the DS task.

### ISD decreases neuronal activity in response to reward cues in the dmPFC

**Figure 3.**
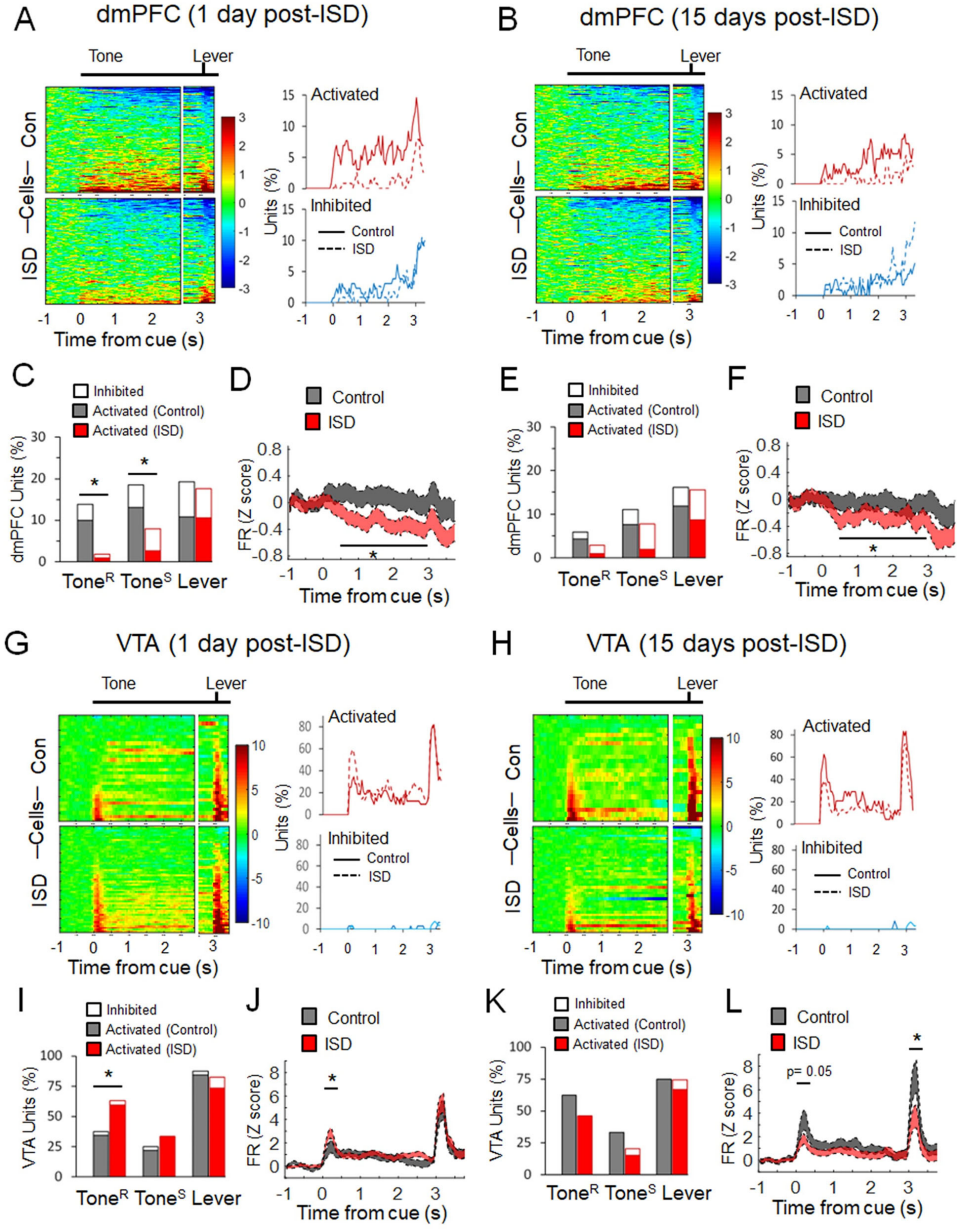
ISD changes the neuronal response to reward cues in the dmPFC and the VTA. ***A***, ***B***, Heat plots represent changes in the baseline-normalized firing rate (*z* scores) for each unit from the dmPFC of control (top, *n* = 7 rats) and ISD (bottom, *n* = 7 rats) in response to cue (Tone) and lever presentation events during DS+ trials, 1 d (***A***; top, *n* = 130 units, bottom, *n* = 114 units) and 15 d (***B***; top, *n* = 118 units, bottom, *n* = 103 units) post-ISD. Each row represents the same as in Figure 2. The line graphs are the percentage of neurons activated and inhibited by these events during DS+ trials. ***C***, ***E***, Bar graphs represent the percentage of dmPFC units significantly activated and inhibited during Tone^R^, Tone^S^, and Lever in DS+ trials for control and ISD, 1 d (***C***) and 15 d (***E***) post-ISD. ISD decreased the number of units responding to Tone^R^ (*χ*^2^_(2)_ = 11.95, *p* = 0.002) and Tone^S^ (*χ*^2^_(2)_ = 8.87, *p* < 0.001), but not Lever (*χ*^2^_(2)_ = 0.18, *p* = 0.911), 1 d post-ISD. ***D***, ***F***, Average population activity (mean ± SEM) in the dmPFC of control and ISD, 1 d (***D***) and 15 d post-ISD (***F***). ISD decreased dmPFC population activity 1 d (*t*_(242)_ = 3.15, *p* = 0.001) and 15 d (*t*_(219)_ = 2.03, *p* = 0.043, independent *t* test, as the average activity in response to Tone^S^) post-ISD. ***G***, ***H***, Heat plots and line graphs represent the same as in ***A***, ***B***, but for each unit from the VTA of control (top, *n* = 6 rats) and ISD (bottom, *n* = 6 rats) in response to cue (Tone) and lever presentation events during DS+ trials, 1 d (***G***; top, *n* = 32 units, bottom, *n* = 57 units) and 15 d (***H***; top, *n* = 24 units, bottom, *n* = 39 units) post-ISD. ***I***, ***K***, Bar graphs represent the percentage of VTA units significantly activated and inhibited during Tone^R^, Tone^S^, and Lever in DS+ trials for control and ISD, 1 d (***I***) and 15 d (***K***) post-ISD. ISD increased the number of units responding to Tone^R^ (*χ*^2^_(1)_ = 5.23, *p* = 0.022), but not Tone^S^ (*χ*^2^_(1)_ = 1.30, *p* = 0.253) or Lever (*χ*^2^_(1)_ = 1.34, *p* = 0.246), 1 d post-ISD. ***J***, ***L***, Average population activity (mean ± SEM) in the VTA of control and ISD, 1 d (***J***) and 15 d post-ISD (***L***). ISD increased VTA population activity during Tone^R^ (*t*_(61)_ = 2.17, *p* = 0.033, Welch's *t* test) 1 d post-ISD, but decreased VTA population activity during Tone^R^ (*t*_(34)_ = 2.01, *p* = 0.050) and Lever (*t*_(44)_ = 2.14, *p* = 0.037, Welch's *t* test) 15 d post-ISD. *p < 0.05 compared with Control.

ISD is a social stress protocol that increases choice impulsivity, reward-seeking behavior, and drug self-administration, which ultimately can facilitate the emergence of addiction-like behavior (Miczek et al., 2008, 2011; Tunstall and Carmack, 2016; Lemon and Del Arco, 2022; Martinez et al., 2024). We focused on reward cues (DS+ trials) and compared both basal activity and neuronal responses during Tone^R^, Tone^S^, and Lever, between control and stressed rats, at two different time points, 1 d post-ISD (short term) and 15 d post-ISD (long term; Fig. 3*A–F*; Tables 1, 2).

As shown in Figure 3*A*,*B*, ISD decreased dmPFC neuronal activity in response to reward cues both 1 and 15 d post-ISD. This decrease was significant at both single units and population activity level. Thus, 1 d post-ISD, stressed animals showed a decreased proportion of activated, but not inhibited, units in response to Tone^R^ (*χ*^2^_(2)_ = 11.95, *p* = 0.002) and Tone^S^ (*χ*^2^_(2)_ = 8.87, *p* < 0.011) compared with controls (Fig. 3*C*, Table 1). In addition, the average population activity during Tone^S^ decreased in stressed compared with control animals (*t*_(242)_ = 3.15, *p* = 0.001, independent *t* test; Fig. 3*D*). Similar effects were observed in 15 d post-ISD. Although the proportion of activated units in response to Tone^R^ (*χ*^2^_(2)_ = 2.23, *p* = 0.32) and Tone^S^ (*χ*^2^_(2)_ = 4.35, *p* = 0.115) were not significantly decreased by ISD (Fig. 3*E*, Table 1), the average population activity during Tone^S^ did decrease in stressed compared with control animals (*t*_(219)_ = 2.03, *p* = 0.043; Fig. 3*F*). ISD did not change the proportion of units that responded to Lever 1 d (*χ*^2^_(2)_ = 0.18, *p* = 0.911) or 15 d post-ISD (*χ*^2^_(2)_ = 1.18, *p* = 0.553). Also, as shown in Table 2, ISD did not change the basal firing rate of neurons, 1 d (*t*_(242)_ = 0.590, *p* = 0.550) or 15 d post-ISD (*t*_(219)_ = 0.140, *p* = 0.880, independent *t* test, as the average of activity during 1 s before the cue). These results indicate that ISD produces a long-term decrease of dmPFC neuronal activity in response to reward cues.

**Table 2.** Baseline firing rates (Hz) of dmPFC and VTA neurons [putative DA/non-DA cells] in control and stressed rats, 1 and 15 d post-ISD

	dmPFC		VTA	
	1 d post-ISD	15 d post-ISD	1 d post-ISD	15 d post-ISD
Control	6.0 ± 0.4 Hz	6.0 ± 0.5 Hz	3.8 ± 0.6 Hz	3.8 ± 0.8 Hz
			[3.3 ± 0.4/6.4 ± 3.2]	[3.6 ± 0.5/5.0 ± 4.1]
ISD	6.4 ± 0.5 Hz	6.1 ± 0.5 Hz	6.0 ± 1.2 Hz	7.9 ± 1.6 Hz*
			[4.0 ± 0.4/12.1 ± 4.7]	[3.4 ± 0.5/14.5 ± 3.2]

Baseline firing rates were calculated using all recorded neurons [dmPFC, Control (1 d = 130; 15 d = 118), ISD (1 d = 114; 15 d = 103); VTA, Control (1 d = 32; 15 d = 24), ISD (1 d = 57; 15 d = 39)].

* *p* < 0.05 compared with Control.

### ISD increases and decreases neuronal activity in response to reward cues in the VTA

Like in the dmPFC, in the VTA, we focus on reward cues (DS+ trials) and compared both basal activity and neuronal responses during Tone^R^, Tone^S^, and Lever, between control and stressed rats, at two different time points, 1 d post-ISD (short term) and 15 d post-ISD (long term; Fig. 3*G–L*; Tables 1, 2). We also classified units as putative dopamine (DA) and nondopamine (non-DA) units (Park and Moghaddam, 2017; Del Arco et al., 2020) to assess whether ISD produced different effects on these VTA subtypes (Fig. 5). This classification is consistent with optogenetically tagged DA neurons observed in previous studies (Cohen et al., 2012; Lohani et al., 2019).

As shown in Figure 3*G*,*H*, ISD produced time-dependent changes in the neuronal response to reward cues during cue and lever presentation events. Thus, 1 d post-ISD, stressed animals showed an increased proportion of activated units in response to Tone^R^ (*χ*^2^_(1)_ = 5.23, *p* = 0.022), but not Tone^S^ (*χ*^2^_(1)_ = 1.30, *p* = 0.253) or Lever (*χ*^2^_(1)_ = 1.34, *p* = 0.246) compared with controls (Fig. 3*I*, Table 1). The average population activity in response to Tone^R^ also increased in stressed animals compared with controls (*t*_(61)_ = 2.17, *p* = 0.033, Welch's *t* test; Fig. 3*J*). In contrast, 15 d post-ISD, the average population activity in response to both Tone^R^ (*t*_(34)_ = 2.01, *p* = 0.050, Welch's *t* test) and Lever (*t*_(44)_ = 2.14, *p* = 0.037, Welch's *t* test) decreased in stressed animals compared with controls (Fig. 3*L*). This decrease in the population activity, however, did not come along with significant changes in the proportion of VTA units responding to these task events [Tone^R^ (*χ*^2^_(1)_ = 1.59, *p* = 0.207), Tone^S^ (*χ*^2^_(1)_ = 2.76, *p* = 0.096); Lever (*χ*^2^_(1)_ = 0.48, *p* = 0.484); Fig. 3*K*, Table 1]. These results indicate that ISD produces time-dependent effects on VTA neuronal activity in response to reward cues.

**Figure 4.**
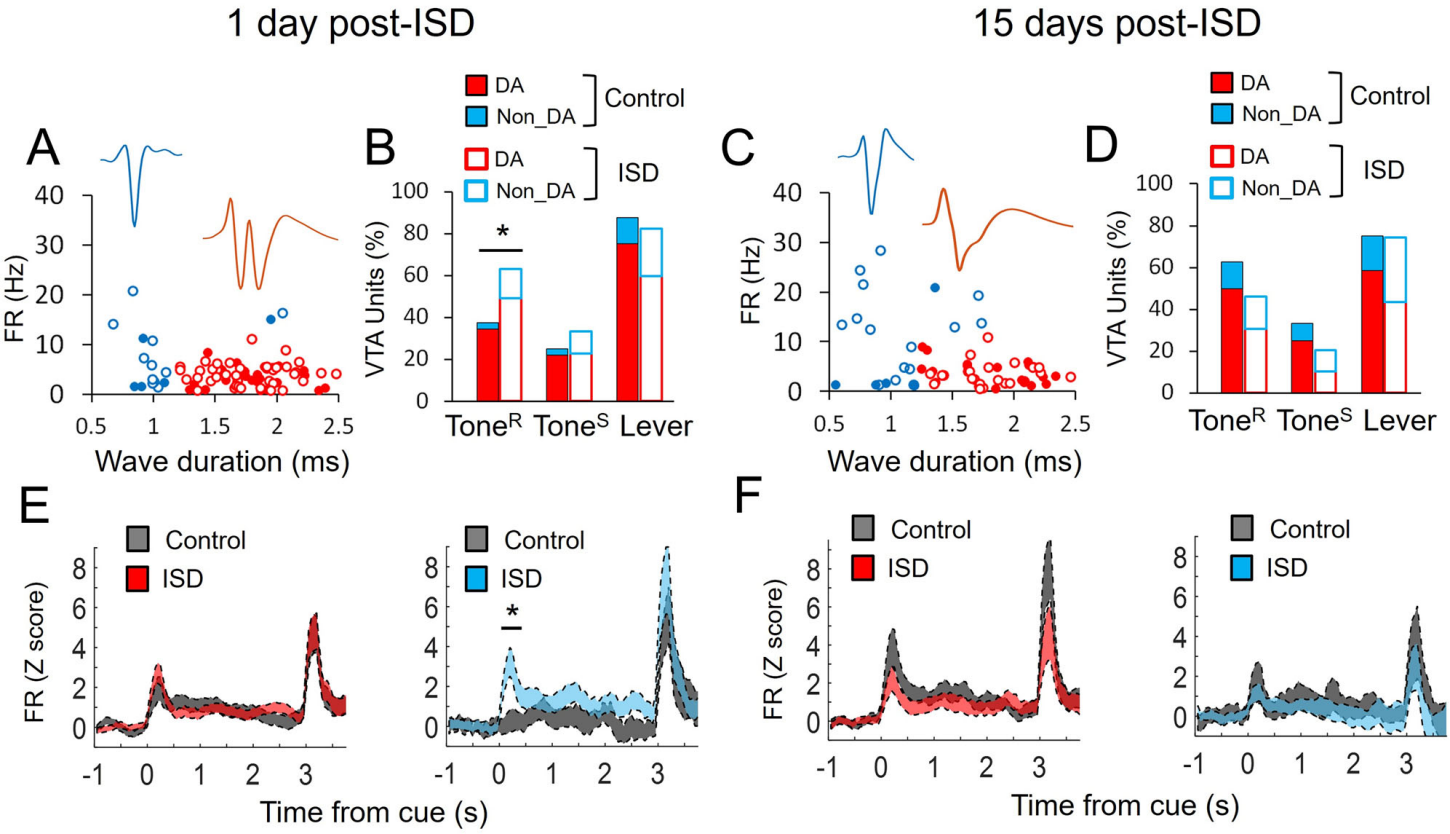
ISD effects on VTA units characterized as putative dopamine (DA) and nondopamine (non-DA) cells. ***A***, ***C***, Electrophysiological characterization of the VTA units shown in this figure as putative DA and non-DA according to their basal firing rate (FR) and wave form duration, in control (*n* = 6 rats) and ISD (*n* = 6 rats), 1 d (***A***) and 15 d (***C***) post-ISD. ***B***, ***D***, Bar graphs represent the percentage of DA and non-DA units significantly activated during Tone^R^, Tone^S^, and Lever in DS+ trials for control and ISD, 1 d (***B***) and 15 d (***D***) post-ISD. ISD increased the number of DA and non-DA units responding to Tone^R^ (*χ*^2^_(2)_ = 6.10, *p* = 0.047), 1 d post-ISD. ***E***, ***F***, Average population activity (mean ± SEM) of DA and non-DA cells in control and ISD, 1 d (***E***) and 15 d post-ISD (***F***). ISD increased the population activity of non-DA cells in response to Tone^R^ (*t*_(13)_ = 3.71, *p* = 0.002, Welch's *t* test), but not DA cells (*t*_(53)_ = 1.35, *p* = 0.180, Welch's *t* test), 1 d post-ISD. *p < 0.05 compared with Control.

Figure 4 shows VTA units from control and stressed rats classified as putative DA and non-DA units according to their firing rate and duration of the action potential (Fig. 4*A*,*C*). Figure 4 also shows the proportion (Fig. 4*B*,*D*) and the average population activity (Fig. 4*E*,*F*) of putative DA and non-DA units in response to Tone^R^, Tone^S^, and Lever, 1 and 15 d post-ISD, respectively. As shown, 1 d post-ISD, both putative DA and non-DA units contributed to increase the proportion of VTA units responding to Tone^R^ in stressed animals compared with controls (*χ*^2^_(2)_ = 6.10, *p* = 0.047; Fig. 4*B*). Also, 1 d post-ISD, the population activity corresponding to putative non-DA units were increased during Tone^R^ in stressed animals compared with controls (non-DA: *t*_(13)_ = 3.71, *p* = 0.002; DA: *t*_(53)_ = 1.35, *p* = 0.180, Welch's *t* test). Fifteen days post-ISD, there were not significant changes in the proportion or population activity corresponding to these VTA neuronal subtypes in stressed animals compared with controls during these task events. Table 2 shows that ISD significantly changed the basal firing rate of VTA neurons 15 d post-ISD (*t*_(54)_ = 2.32, *p* = 0.024, Welch's *t* test), but not 1 d post-ISD (*t*_(78)_ = 1.65, *p* = 0.102, Welch's *t* test). However, the basal firing rate of putative DA and non-DA units from control and stressed animals was not statistically different 1 d (*t*_(62)_ = 1.35, *p* = 0.179, and *t*_(16)_ = 1.04, *p* = 0.310, respectively) or 15 d post-ISD (*t*_(38)_ = 0.20, *p* = 0.84, and *t*_(10)_ = 1.95, *p* = 0.078, respectively, Welch's *t* test).

### ISD differently changes discrimination accuracies in the dmPFC and the VTA

**Figure 5.**
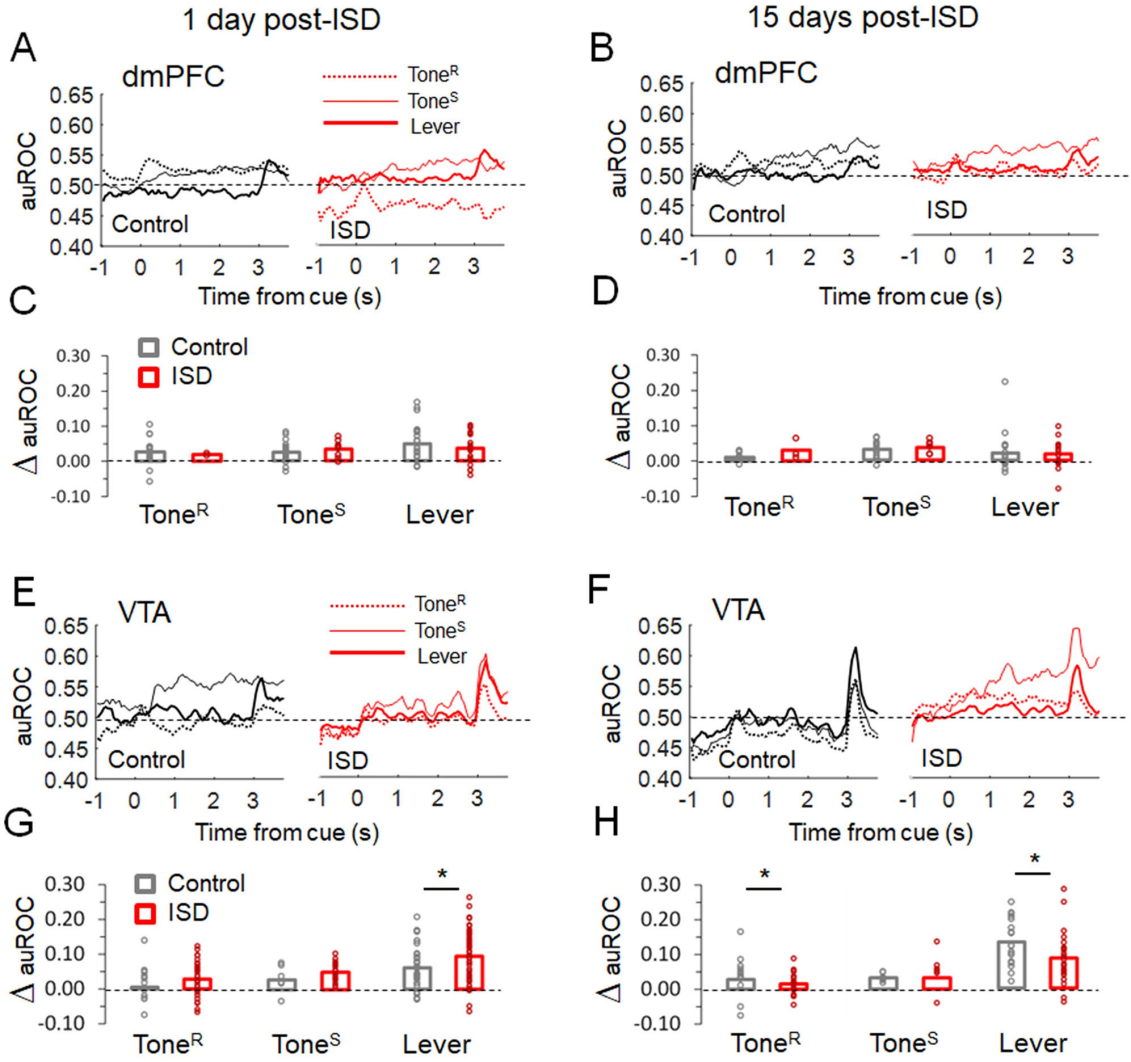
ISD modulates the selectivity of VTA, but not dmPFC, units for reward cues. ***A***, ***B***, Temporal profile of average auROC values for responsive neurons in the dmPFC during Tone^R^, Tone^S^, and Lever, 1 d (***A***) and 15 d (***B***) post-ISD. ***C***, ***D***, Bars represent the average increase of auROC (compared with baseline) in the dmPFC for units responsive during Tone^R^, Tone^S^, and Lever in control (*n* = 7 rats) and ISD (*n* = 7 rats), 1 d (***C***) and 15 d (***D***) post-ISD. ***E***, ***F***, Temporal profile of the average auROC values for responsive neurons in the VTA during Tone^R^, Tone^S^, and Lever, 1 d (***E***) and 15 d (***F***) post-ISD. ***G***, ***H***, Bars represent the average increase of auROC (compared with baseline) in the VTA for units responsive during Tone^R^, Tone^S^, and Lever in control (*n* = 6 rats) and ISD (*n* = 6 rats), 1 d (***G***) and 15 d (***H***) post-ISD. Dots represent individual single units's auROC increases. *p < 0.05 compared with Control, after Welch's *t* test.

**Figure 6.**
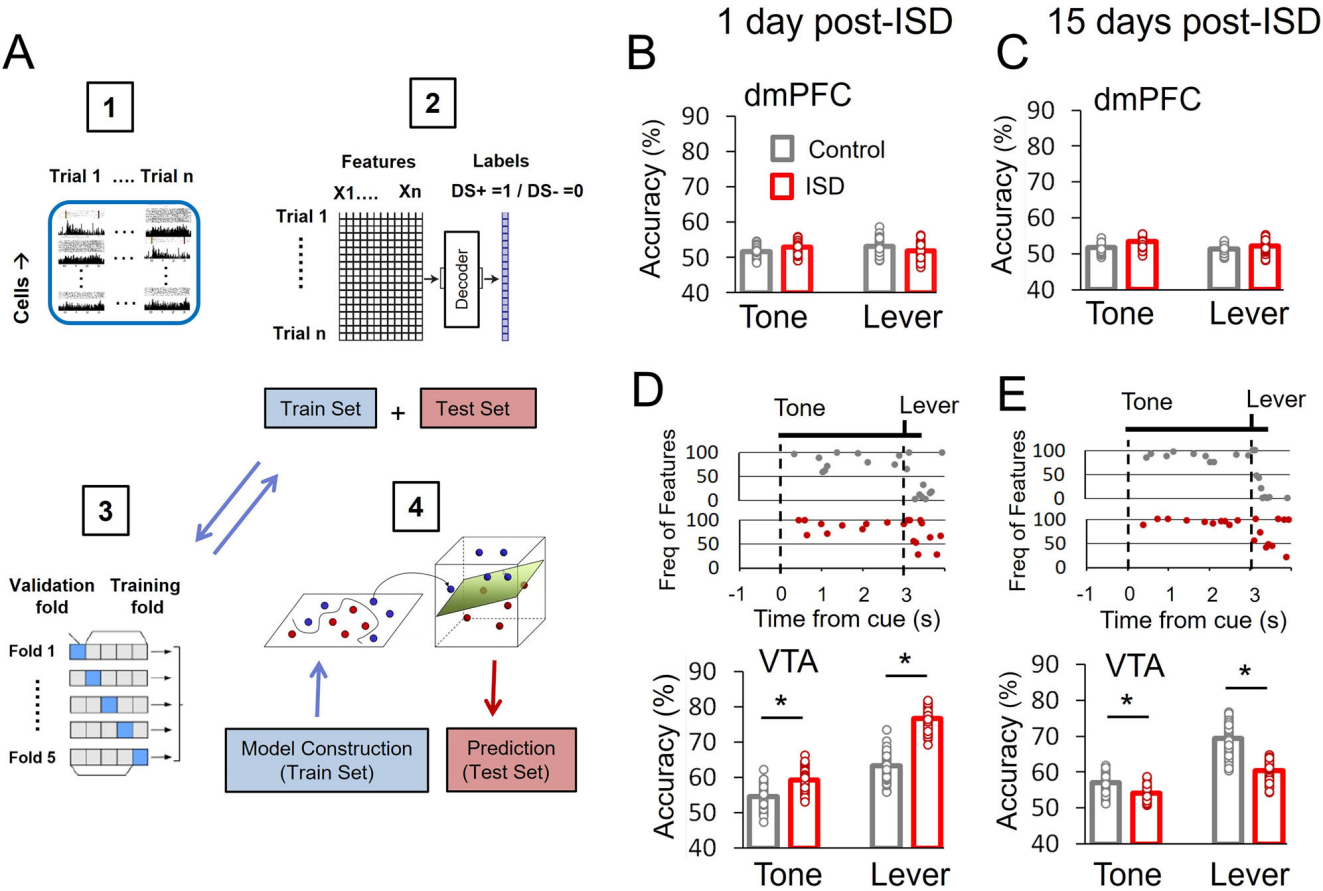
ISD differently modulates decoding accuracy of neuronal populations in the dmPFC and the VTA. ***A***, Diagram depicting the decoding procedure. (1) For each cell at a given trial, the population activity was aligned around the cue onset time and extended to 1 s after the lever presentation (from −1 to 4 s). An example dataset from one animal consisting of *n* trials is shown. (2) Time stamps of average population activity for each trial were used as features to discern when neuronal populations better predicted the value of the cue. Each trial was given a label based on the value of the cue, 1 for DS+ and 0 for DS−. Then data across all animals and trials were combined to construct a full dataset with *n* features (100 features = 100 of 50 ms time bins) and *n* labels, where *n* was the number of all the trials across animals in each session (50 DS+ and 50 DS− trials per animal). (3) Next, the full dataset was split into training and testing sets and hyperparameters of the decoder optimized using a fivefold cross-validation within the training set. (4) After optimization, the decoder was retrained based on the full training set. Finally, the trained model was applied on testing sets to obtain decoding accuracy. This process was repeated 100 times, using different training sets, and the 10 most frequent features were selected to calculate mean accuracies for cue (Tone) and lever presentation (Lever) events during the task. ***B***, ***C***, Decoding accuracy in the dmPFC for control (*n* = 7 rats) and ISD (*n* = 7 rats), 1 d (***B***) and 15 d (***C***) post-ISD. ***D***, ***E***, Frequency of the best 10 selected features (top) and decoding accuracy (bottom) in the VTA for control (*n* = 6 rats) and ISD (*n* = 6 rats), 1 d (***D***) and 15 d (***E***) post-ISD. *p < 0.05 compared with Control, after independent *t* test.

We evaluated how accurately cue- and lever presentation-evoked neuronal activity in the dmPFC and the VTA of control and stressed animals encoded the value of cues during the DS task, both 1 and 15 d post-ISD. First, we computed auROC curves from distribution of firing rates during DS+ versus DS− trials (Wallis and Miller, 2003; Kim et al., 2010; Del Arco et al., 2017; Table 3, Fig. 5). This approach focused on single units responsive to Tone^R^, Tone^S^, and Lever task events. Second, given that the activity of the entire neuronal population is likely to contain relevant task-related information, we also performed a decoding analysis to determine trial discrimination accuracy of neuronal population activity associated to these task events (Fig. 6).

**Table 3.** Proportion of units that discriminate reward from nonreward cues (DS+ vs DS−; i.e., selective units) according to their auROC values during the tone and lever events in the dmPFC and the VTA, 1 and 15 d post-ISD

Control	dmPFC		VTA	
	1 d post-ISD	15 d post-ISD	1 d post-ISD	15 d post-ISD
Tone^R^	7% (4%)	5% (5%)	7% (5%)	13% (7%)
Tone^S^	6% (4%)	5% (4%)	7% (3%)	8% (4%)
Lever	11% (4%)*	8% (4%)	27% (5%)*	41% (7%)*
ISD				
Tone^R^	5% (4%)	5% (4%)	15% (6%)	7% (3%)
Tone^S^	5% (4%)	4% (3%)	9% (5%)	11% (4%)
Lever	8% (4%)	7% (4%)	39% (7%)*	30% (6%)*

In parenthesis the proportion of units after shuffling the order of trials.

* *p* < 0.05 compared with shuffled.

Table 3 shows the proportion of units that discriminated DS+ from DS− (i.e., selective units) according to experimental auROC compared with shuffled auROC values (see Materials and Methods), in the dmPFC and the VTA, for control and stressed animals, both 1 and 15 d post-ISD. In the dmPFC, a significant proportion of units discriminated reward from nonreward cues during Lever in control animals, 1 d post-ISD (11%, *χ*^2^_(1)_ = 4.59, *p* = 0.031). In the VTA, a significant proportion of units discriminated reward cues during Lever in control animals, 1 d post-ISD (27%, *p* < 0.05, Fisher exact test) and 15 d post-ISD (41%, *p* < 0.05), and also in stressed animals, 1 d post-ISD (39%, *p* < 0.001, Fisher exact test) and 15 d post-ISD (30%, *p* < 0.05). ISD did not change the proportion of selective units in the dmPFC or the VTA 1 or 15 d post-ISD.

Figure 5 shows auROC values for responsive units (i.e., temporal profile and increases compared with baseline) in the dmPFC (Fig. 5*A–D*) and the VTA (Fig. 5*E–H*), for control and stressed animals, both 1 and 15 d post-ISD. In the dmPFC of control animals, auROC average values of responsive units significantly increased after both cue and lever presentation events, 1 and 15 d post-ISD (1 d: Tone^R^: *t*_(25)_ = 6.36, *p* < 0.001; Tone^S^: *t*_(27)_ = 6.76, *p* < 0.001; Lever: *t*_(27)_ = 5.83, *p* < 0.001; 15 d: Tone^R^: *t*_(13)_ = 4.37, *p* < 0.001; Tone^S^: *t*_(13)_ = 5.71, *p* < 0.001; Lever: *t*_(22)_ = 2.25, *p* = 0.034, paired *t* test, compared with baseline auROC; Fig. 5*C*,*D*). However, as also shown, ISD did not change these auROC average increases in the dmPFC. In the VTA of control animals, the average auROC values of responsive units significantly increased after both cue and lever presentation events, 1 and 15 d post-ISD (1 d: Tone^R^: *t*_(13)_ = 2.83, *p* = 0.014; Tone^S^: *t*_(7)_ = 4.07, *p* = 0.004; Lever: *t*_(27)_ = 5.22, *p* < 0.001; 15 d: Tone^R^: *t*_(14)_ = 6.76, *p* < 0.001; Tone^S^: *t*_(8)_ = 6.93, *p* = 0.012; Lever: *t*_(17)_ = 9.01, *p* < 0.01, paired *t* test, compared with baseline auROC; Fig. 5*G*,*H*). Unlike dmPFC, ISD did change auROC average increases. Specifically, auROC average increases during Lever were significantly higher 1 d post-ISD (*t*_(58)_ = 2.30, *p* = 0.025, Welch's *t* test) and lower 15 d post-ISD (*t*_(42)_ = 2.55, *p* = 0.014, Welch's *t* test), in stressed animals compared with controls. Also, auROC increases were significantly lower during Tone^R^ 15 d post-ISD (*t*_(28)_ = 3.45, *p* = 0.002, Welch's *t* test) in stressed animals compared with controls. Overall, the auROC analysis indicates that neurons responding to lever presentation in the VTA, compared with the dmPFC, are more accurate discriminating reward cues and that ISD changes discrimination accuracy of VTA, but not dmPFC, single neurons in a time-dependent manner.

Figure 6 shows a diagram of the machine learning approach utilized to assess decoding accuracies of dmPFC and VTA population activities (Fig. 6*A*). We utilized the regression logistic model LASSO (Least Absolute Shrinkage and Selection Operator) due to its ability to select the features that were most useful to the model's prediction (Akalin, 2020; Koban et al., 2023). Decoding accuracies were computed considering the 10 most frequent features selected by the model (see Materials and Methods). Figure 6 also shows decoding accuracies for the dmPFC (Fig. 6*B*,*C*) and the VTA (Fig. 6*D*,*E*, bottom) populations of control and stressed animals after the cue and lever presentation events, 1 and 15 d post-ISD. As shown, decoding accuracies for dmPFC activity were at chance level (50%) for both groups of animals and sessions. In contrast, decoding accuracies for VTA activity were higher than chance level and significantly different between stressed and control animals. Specifically, in the VTA, decoding accuracies after the cue onset were significantly higher 1 d post-ISD (59% ISD vs 55% control, *t*_(198)_ = 12.62, *p* < 0.001) and lower 15 d post-ISD (54% ISD vs 57% control, *t*_(198)_ = 10.33, *p* < 0.001, independent *t* test), in stressed animals compared with controls. Similar effects were observed during the lever presentation at 1 d post-ISD (77% ISD vs 63% control, *t*_(198)_ = 33.71, *p* < 0.001) and 15 d post-ISD (60% ISD vs 69% control, *t*_(198)_ = 23.29, *p* < 0.001, independent *t* test). Figure 6, *D* and *E*, top, shows the frequency of the best 10 features extracted by the model across 50 ms time bins in both groups of rats during the two sessions. These results indicate that the VTA neuronal population, compared with the dmPFC, predicts more accurately the value of cues during the DS task, especially during the lever presentation event. They also indicate that ISD changes discrimination accuracy of the VTA neuronal population in a time-dependent manner. Table 4 shows a summary of statistical results according to brain area, data analyzed, and test used.

**Table 4.** Summary of statistical results according to brain area, data analyzed, and test used

	Brain area	Data analyzed	Type of test	Statistical results
Behavior (Fig. 1)		Training (1–5) (DS+ vs DS−)	Three-way ANOVA	(trial type × session)
		# Lever Presses		*F*_(4,40)_ = 31.25, *p* < 0.001
		Latency to Press		*F*_(4,24)_ = 8.78, *p* < 0.001
		Post-training (S1–S5) (Control vs ISD)		(trial type × session × group)
		# Lever presses		*F*_(4,52)_ = 0.47, *p* = 0.758
		Latency to press		*F*_(4,40)_ = 0.17, *p* = 0.954
Neuronal encoding (Fig. 3)	dmPFC	1 d post-ISD (Control vs ISD)		
		# Units	Chi square	
		Tone^R^		*Χ*^2^_(2)_ = 11.95, *p* = 0.002; phi = 0.77
		Tone^S^		*χ*^2^_(2)_ = 8.87, *p* = 0.011; phi = 0.52
		Lever		*χ*^2^_(2)_ = 0.18, *p* = 0.911
		Population activity	Independent *t* test	
		Tone^S^		*t*_(242)_ = 3.15, *p* = 0.001; *d* = 0.40
		15 d post-ISD		
		# Units	Chi square	
		Tone^R^		*Χ*^2^_(2)_ = 2.23, *p* = 0.320
		Tone^S^		*χ*^2^_(2)_ = 4.35, *p* = 0.115
		Lever		*χ*^2^_(2)_ = 1.18, *p* = 0.553
		Population activity	Independent *t* test	
		Tone^S^		*t*_(219)_ = 2.03, *p* = 0.043; *d* = 0.27
	VTA	1 d post-ISD (Control vs ISD)		
		# Units	Chi square	
		Tone^R^		*Χ*^2^_(1)_ = 5.23, *p* = 0.022; phi = 0.34
		Tone^S^		*χ*^2^_(1)_ = 1.30, *p* = 0.253
		Lever		*χ*^2^_(1)_ = 1.34, *p* = 0.246
		Population activity	Welch'*s t* test	
		Tone^R^		*t*_(61)_ = 2.17, *p* = 0.033; *d* = 0.55
		15 d post-ISD		
		# Units	Chi square	
		Tone^R^		*Χ*^2^_(1)_ = 1.59, *p* = 0.207
		Tone^S^		*χ*^2^_(1)_ = 2.76, *p* = 0.096
		Lever		*χ*^2^_(1)_ = 0.48, *p* = 0.484
		Population activity	Welch'*s t* test	
		Tone^S^		*t*_(34)_ = 2.01, *p* = 0.050; *d* = 0.69
		Lever		*t*_(44)_ = 2.14, *p* = 0.037; *d* = 0.65
Discriminative accuracy (Figs. 5, 6)	dmPFC	1 d post-ISD (Control vs ISD)		
		Units (auROC)	Welch'*s t* test	
		Tone^R^		*t*_(17)_ = 0.49, *p* = 0.627
		Tone^S^		*t*_(18)_ = 1.09, *p* = 0.287
		Lever		*t*_(48)_ = 0.94, *p* = 0.352
		15 d post-ISD		
		Units (auROC)	Welch'*s t* test	
		Tone^R^		*t*_(7)_ = 1.14, *p* = 0.29
		Tone^S^		*t*_(25)_ = 0.70, *p* = 0.49
		Lever		*t*_(35)_ = 0.08, *p* = 0.93
	VTA	1 d post-ISD (Control vs ISD)		
		Units (auROC)	Welch'*s t* test	
		Tone^R^		*t*_(20)_ = 0.65, *p* = 0.523
		Tone^S^		*t*_(15)_ = 0.60, *p* = 0.553
		Lever		*t*_(58)_ = 2.30, *p* = 0.025; *d* = 0.60
		Population (LASSO)	Independent *t* test	
		Tone		*t*_(198)_ = 12.62, *p* < 0.001; *d* = 1.80
		Lever		*t*_(198)_ = 33.71, *p* < 0.001; *d* = 4.79
		15 d post-ISD	Welch'*s t* test	
		Units (auROC)		
		Tone^R^		*t*_(28)_ = 3.45, *p* = 0.002; *d* = 1.30
		Tone^S^		*t*_(8)_ = 2.02, *p* = 0.074; *d* = 1.43
		Lever		*t*_(42)_ = 2.55, *p* = 0.014; *d* = 0.79
		Population (LASSO)	Independent *t* test	
		Tone		*t*_(198)_ = 10.33, *p* < 0.001; *d* = 1.47
		Lever		*t*_(198)_ = 23.29, *p* < 0.001; *d* = 3.31

## Discussion

ISD is a social stress protocol that increases choice impulsivity and drug self-administration up to several weeks after the termination of stress, which can facilitate the emergence of addiction-like behavior (Miczek et al., 2008, 2011; Tunstall and Carmack, 2016; Lemon and Del Arco, 2022; Martinez et al., 2024). We examined in vivo neuronal dynamics during a reward-seeking task and found that ISD differentially disrupts the neuronal encoding of reward cues in the dmPFC and the VTA. These effects spanned single neurons and neuronal populations and were dependent on time after stress. In the dmPFC, ISD decreased cue-evoked neuronal activity 1 and 15 d post-ISD. In the VTA, ISD both increased (1 d post-ISD) and decreased (15 d post-ISD) cue-evoked neuronal activity and reward discrimination accuracy. Despite the lack of changes in behavioral performance, these results suggest that ISD degrades outcome anticipation-related processing in the dmPFC and modulates incentive salience in the VTA, providing novel insights into the neurofunctional signatures underlying vulnerability to substance abuse after stress.

### dmPFC and VTA neurons differentially encode reward cues during the DS task

In the dmPFC, we found that different populations of neurons were activated and inhibited in response to cues that predicted the possibility of earning (DS+), or not (DS−), rewards (i.e., sugar pellets; Moorman and Aston-Jones, 2015; Otis et al., 2017; Spring et al., 2021b). We identified neurons that responded immediately to cue onset and lever presentation and neurons that displayed a sustained response after cue onset. Importantly, cue-evoked and lever presentation-evoked neuronal populations did not substantially overlap, which suggests that different dmPFC neurons encode reward-related features associated with cue and lever events such as outcome anticipation and incentive salience, respectively (Moorman and Aston-Jones, 2015; Del Arco et al., 2017; Otis et al., 2017; Munkhzaya et al., 2020; Laubach et al., 2021). Notably, a significantly higher number of dmPFC neurons responded to both cue (i.e., sustained activity) and lever presentation during DS+ compared with DS− trials, which supports the role of these neurons in processing anticipation and motivation toward the upcoming reward. In contrast to single neurons, dmPFC average population activity did not change between DS+ and DS− trials, which suggests that the population activity does not differently encode reward and nonreward cues during the task. In addition, ROC and decoding analysis showed that single neurons and population activity have a limited contribution to reward discrimination, which suggests that dmPFC neurons do not play a significant role driving DS task performance (Moorman and Aston-Jones, 2015).

In the VTA, and in contrast to dmPFC, cue-evoked and lever presentation-evoked neuronal populations did overlap which suggests that the same VTA neurons signal both cue and lever events. VTA-evoked neuronal activity (i.e., single neurons and population) was higher during DS+ compared with DS− trials and showed a high degree of reward discrimination. These results support the well-established role of VTA neurons, especially dopamine cells, in salience attribution and incentive motivation (Wyvell and Berridge, 2001; Flagel et al., 2011; Robinson et al., 2013; Halbout et al., 2019; Goedhoop and Arbab, 2023; Iglesias et al., 2023; Stuber, 2023), and suggest that VTA neurons contribute to DS task performance (Halbout et al., 2019; Goedhoop and Arbab, 2023). In the current study, we also grouped VTA neurons into putative dopamine (DA) and nondopamine (non-DA) cells according to the shape and wavelength of the action potential (Park and Moghaddam, 2017; Del Arco et al., 2020) and show that both DA and non-DA cells respond to cue and lever events during both DS+ and DS− trials.

### ISD degrades outcome anticipation in the dmPFC

ISD decreased cue-evoked neuronal responses, in the proportion of activated neurons and/or in average population activity, 1 and 15 d after the last stress episode, demonstrating a long-term disruption of reward encoding in the dmPFC. Critically, ISD altered cue-evoked neuronal activation, but not inhibition. A recent study provides evidence that dmPFC neurons activated and inhibited by reward cues belong to two nonoverlapping populations that correspond to dmPFC→nucleus accumbens and dmPFC→paraventricular nucleus of the thalamus projecting neurons, respectively, and play different roles encoding reward predicting cues (Otis et al., 2017). Considering this study, our results suggest that ISD degrades outcome anticipation-related processing by decreasing the activation of dmPFC→nucleus accumbens projecting neurons in response to reward cues, which could be linked to alterations in choice, value, and performance monitoring, among other functions (Del Arco et al., 2017; Otis et al., 2017; Rikhye et al., 2018; Laubach et al., 2021; Bercovici et al., 2023). In support of this possibility, we have shown that ISD increases choice impulsivity (Martinez et al., 2024), which is a value-based decision-making deficit that has been associated with dmPFC neuronal activity (Sackett et al., 2019) and specifically with a decreased activation of dmPFC→nucleus accumbens projecting neurons (Wenzel et al., 2023).

In contrast to cue-evoked neuronal responses, ISD did not change lever presentation-evoked neuronal activation in the dmPFC, indicating that the incentive salience linked to the action required to earn rewards is unaltered in stressed animals (i.e., action–outcome association; Simon et al., 2015). This presumably lack of changes in the prefrontal representation of action–outcome associations could explain, in part, the lack of performance changes in the DS task after ISD. Overall, our data suggests that the effects of ISD disrupting reward encoding in the dmPFC are specific of neuronal population (i.e., dmPFC→nucleus accumbens neurons) and reward encoding feature (i.e., outcome anticipation) and predispose individuals to seek out rewards after stress.

### ISD increases and decreases the sensitivity of VTA neurons to reward cues

In the VTA, previous studies have shown that chronic stress increases (chronic social defeat) or decreases (chronic unpredictable stress) basal activity of VTA dopamine neurons (Hollon et al., 2015; Baik, 2020; Douma and de Kloet, 2020) and have associated these changes with depression-related behaviors (i.e., anhedonia) and, ultimately, reduced motivation for rewards in susceptible animals (Krishnan et al., 2007; Cao et al., 2010; Tye et al., 2012; Chaudhury et al., 2013; Friedman et al., 2014; Zhong et al., 2018; Zhang et al., 2024). Unlike previous studies, we focused on cue-evoked neuronal activity and utilized an animal model of repeated stress that increases motivation for rewards. We found that ISD increased cue-evoked neuronal activity and reward value discrimination accuracy at both single neuron and neuronal population levels 1 d after stress. These results demonstrate an enhanced sensitivity of VTA neurons to reward cues and suggest that reward cues are more salient for stressed (vulnerable) animals compared with controls 1 d after stress. This interpretation fits well with previous studies that link enhanced activation of the mesolimbic dopamine system to increased incentive salience and reward-seeking behavior (Beckmann et al., 2011; Robinson et al., 2013; Boyson et al., 2014; Koob and Schulkin, 2019).

Importantly, unlike dmPFC, the effects of ISD in the VTA were dependent on time after stress. Thus, ISD decreased both cue- and lever presentation-evoked population neuronal activity as well as discrimination accuracy 15 d after the last stress episode. These results suggest decreased incentive salience in response to reward cues in stressed animals compared with controls and points to decreased motivation for rewards 15 d after stress. However, this possibility contrasts with the well-established increased reward-seeking and drug self-administration behavior demonstrated after ISD (Miczek et al., 2008, 2011; Boyson et al., 2014; Tunstall and Carmack, 2016; Lemon and Del Arco, 2022). Alternatively, it is possible that the stress-induced decreased sensitivity of VTA neurons shown here reflects reward deficiency, which might be consistent with increased reward-seeking in the long term (Koob, 2013; Leyton, 2014; Koob and Schulkin, 2019). Further studies will be required to substantiate this possibility. Our analysis also characterized putative dopamine and nondopamine cells to show that ISD did not change the basal firing rate of dopamine cells and that both dopamine and nondopamine cells contributed to evoked activity changes produced by ISD in the short and long term.

### ISD did not change behavioral performance

As shown, ISD disrupted the neuronal encoding of reward cues, but these effects were not correlated with changes in DS task performance, which could challenge the interpretation of these results in the context of reward-seeking behavior. However, in a recent study using the same stress protocol, we have shown that ISD alters value-based reward-seeking behavior and increases choice impulsivity (Martinez et al., 2024), which indicates that ISD alters specific neurocognitive processes that regulate reward-seeking behavior (i.e., decision-making, subjective evaluation, incentive motivation) but not others (i.e., stimulus discrimination). Overall, our results demonstrate for the first time that ISD disrupts how the brain processes reward cues at the neuronal level, which is relevant in the context of stress-induced vulnerability.

### Limitations

This study has limitations. First, we only used male rats. Previous studies have shown that ISD produces larger and longer lasting effects on drug self-administration and behavioral sensitization in female compared with male rats (Holly et al., 2012). Based on our results in males, we speculate a larger and longer lasting disruption of reward encoding in the dmPFC and the VTA of females compared with males. Yet, future studies are required to substantiate this speculation. Second, we did not accurately identify cell types or neuronal projections in the dmPFC or the VTA. In the dmPFC, we did find that ISD decreased the activity of cue-activated cells but not cue-inhibited cells which is consistent with changes in the activity of specific dmPFC efferent projections (Otis et al., 2017) after ISD. In the VTA, we find that ISD affected similarly both putative DA and non-DA cells. Yet, we acknowledge that this characterization, although consistent with previous studies (Cohen et al., 2012; Lohani et al., 2019), is not perfectly accurate and, therefore, our observations regarding the role of VTA DA and non-DA cells in reward encoding are limited.

### Novelty and clinical relevance

**Figure 7.**
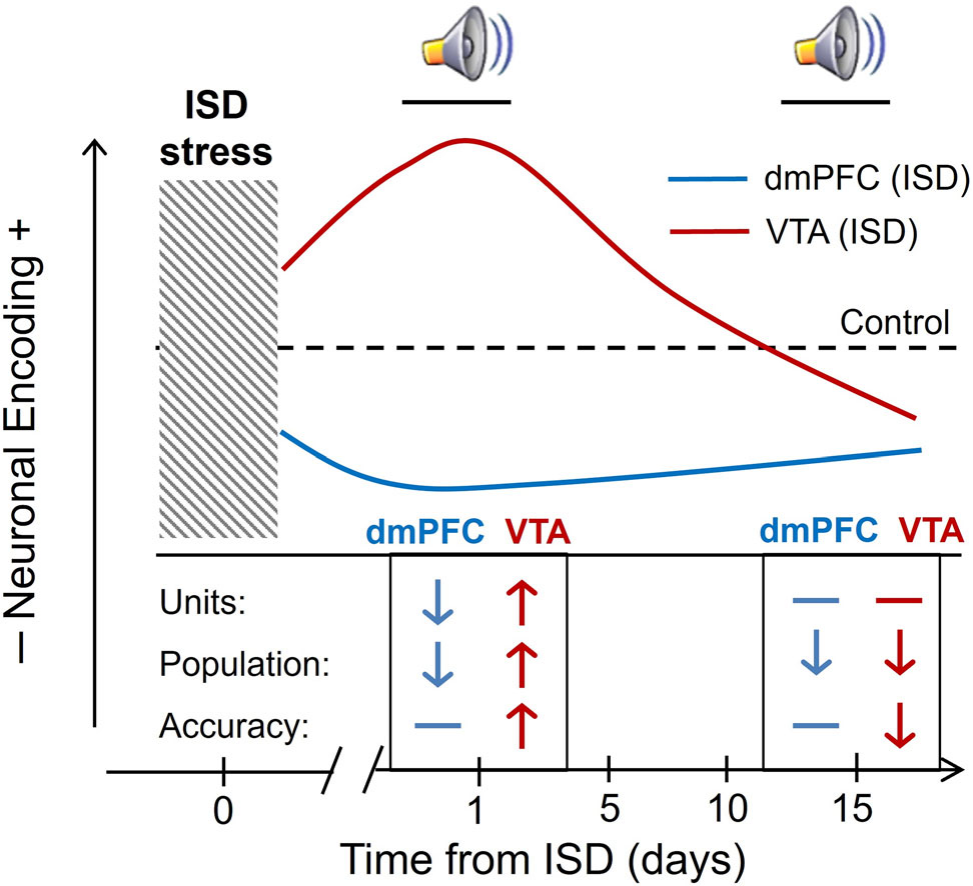
Summary of main findings. ISD stress differently disrupts neuronal dynamics of reward encoding in the dmPFC and the VTA. The line graph is an ideal representation of the observed cue-evoked neuronal activity changes (i.e., neuronal encoding) produced by ISD, compared with Control (dashed line), 1 and 15 d after ISD. The graph includes a table (bottom) that shows the direction of the neuronal changes in both areas of the brain 1 and 15 d after stress, considering Units (% change of single units), Population (average population activity), and Accuracy (discrimination accuracy). The graph highlights the hypothesis that stress-induced vulnerability to substance abuse is a dynamic state that involves time-dependent disruptions of different reward encoding features in the dmPFC (i.e., outcome anticipation-related processing) and the VTA (i.e., salience attribution; see text).

Previous studies in humans and animal models suggest that changes in the brain response to reward cues increase vulnerability to develop substance use disorders and that subtle changes in cognition and motivation support the initiation and escalation of substance consumption (Goldstein and Volkow, 2011; Sturman and Moghaddam, 2011; Courtney et al., 2015; Volkow and Morales, 2015; Kim et al., 2016; Guillem and Ahmed, 2017; Zilverstand et al., 2018). Our study, which simultaneously and longitudinally records the neuronal response to reward cues in the dmPFC and the VTA, provides novel insights into the neuronal dynamics that underlie higher risk of developing substance abuse after stress. It demonstrates that a history of episodic social stress differently disrupts reward encoding in the dmPFC and the VTA and suggests that stress-induced vulnerability is not a static state but rather a dynamic state where a degraded outcome anticipation processing (dmPFC-related) develops together with both increased and decreased incentive salience (VTA-related) overtime (Fig. 7). Based on the existing literature, these neuronal encoding disruptions are consistent with a higher predisposition to seek out rewards that might be expressed through different cognitive adaptations after stress (i.e., value-based decision-making deficits, enhanced salience attribution, reward deficiency; Miczek et al., 2008; Logrip et al., 2012; Robinson et al., 2013; Tunstall and Carmack, 2016). Future studies will determine how these neuronal and behavioral mechanisms orchestrate the transition from stress to substance use disorders.

## Synthesis

Reviewing Editor: Sam Golden, The University of Washington Decisions are customarily a result of the Reviewing Editor and the peer reviewers coming together and discussing their recommendations until a consensus is reached. When revisions are invited, a fact-based synthesis statement explaining their decision and outlining what is needed to prepare a revision will be listed below. The following reviewer(s) agreed to reveal their identity: NONE. Note: If this manuscript was transferred from JNeurosci and a decision was made to accept the manuscript without peer review, a brief statement to this effect will instead be what is listed below.

Dear Dr. Del Arco,

I have reviewed the comments from your manuscripts review at JN, as well as the full rebuttal and revised manuscript. I find your revisions to be appropriate for publication in eNeuro without further review. Thank you for the revised submission and congratulations.
